# Multimode Resource-Constrained Multiple Project Scheduling Problem under Fuzzy Random Environment and Its Application to a Large Scale Hydropower Construction Project

**DOI:** 10.1155/2014/463692

**Published:** 2014-01-15

**Authors:** Jiuping Xu, Cuiying Feng

**Affiliations:** ^1^State Key Laboratory of Hydraulics and Mountain River Engineering, Sichuan University, Chengdu 610064, China; ^2^Uncertainty Decision-Making Laboratory, Sichuan University, Chengdu 610064, China

## Abstract

This paper presents an extension of the multimode resource-constrained project scheduling problem for a large scale construction project where multiple parallel projects and a fuzzy random environment are considered. By taking into account the most typical goals in project management, a cost/weighted makespan/quality trade-off optimization model is constructed. To deal with the uncertainties, a hybrid crisp approach is used to transform the fuzzy random parameters into fuzzy variables that are subsequently defuzzified using an expected value operator with an optimistic-pessimistic index. Then a combinatorial-priority-based hybrid particle swarm optimization algorithm is developed to solve the proposed model, where the combinatorial particle swarm optimization and priority-based particle swarm optimization are designed to assign modes to activities and to schedule activities, respectively. Finally, the results and analysis of a practical example at a large scale hydropower construction project are presented to demonstrate the practicality and efficiency of the proposed model and optimization method.

## 1. Introduction

In scheduling problem, the resource-constrained project scheduling problem (RCPSP) is a classical well-known problem where the activities of a project must be scheduled to minimize its project duration under the presence of precedence and resource constraints. As a special extension of the RCPSP, the multimode resource-constrained project scheduling problem (MRCPSP) has emerged and has been addressed by many researchers [1–4], where each activity can be executed in one of several modes representing a relationship between the resource requirements of the activity and its duration [5]. Within the classical MRCPSP, most research considers project management in terms of a single project, but due to the complexity and natural diversification of a large scale project, there is growing interest in the multimode resource-constrained multiple project scheduling problem (MRCMPSP). While many studies [6–8] have made a significant contribution to multiple project scheduling, they have not considered multimode selection, nor discussed its application to large scale hydropower construction projects. Project cost and time are crucial aspects of construction project management and have received significant attention [9, 10]. As another typical focus in project management, project quality needs to be taken into account when solving the MRCMPSP. With these issues in mind, this paper focuses on a time/cost/quality trade-off (TCQT) optimization for the MRCMPSP, that is, minimizing the weighted project makespan and project cost and maximizing project quality under the presence of precedence and resource constraints in multiple parallel projects with multimode for each activity.

In practice, the MRCMPSP is often complex with inevitably encountering uncertainty because of unforeseen factors such as the changing weather, labor inefficiency, changing markets, and construction technology. Though previous researches [11–14] have successfully used probability theory to address the uncertainties in duration and cost when solving project scheduling problem, sometimes the probability distributions for uncertain parameters may be unknown or just partially known because of a lack of statistical data. In this case, fuzzy set theory may be more appropriate than random variables to describe uncertain parameters. First proposed by Zadeh [15] and consequently developed by researchers such as Dubois and Prade [16], fuzzy theory has been a useful tool in dealing with ambiguous information [17, 18]. Prade [19] first applied fuzzy set theory to the project scheduling problem and from then on many papers [20–22] have been devoted to an RCPSP under a fuzzy environment. While these studies have significantly improved the uncertainty in the RCPSP, they are incapable of reflecting hybrid uncertainty where fuzziness and randomness coexist. For MRCMPSP in large scale construction projects, imprecision and complexity are usually hybrid uncertain and cannot be dealt with using simple fuzzy logic or random logic. In this case, fuzzy random variables, introduced by Kwakernaak [23, 24] and Kruse and Meyer [25], can be employed because they are able to deal with the two types of uncertainty simultaneously. This approach has been recommended by many scholars and encouraged further research into uncertain events [26–28]. With these studies in mind, fuzzy random uncertainty is adopted in this paper to describe the hybrid uncertain environment for MRCMPSP in large scale construction project.

With the MRCMPSP being intrinsically difficult and the model being nonlinear, nonconvex, and nondifferentiable, traditional exact scheduling methods, such as PERT (program evaluation and review technique) and CPM (critical path method), are not suitable for such scheduling problems; see Brucker et al. [29], Potts and Kovalyov [30], and Detti [31]. Thus, effort has been made to develop effective heuristic algorithms to solve the RCPSP, such as genetic algorithms [32, 33], simulated annealing [3], particle swarm optimization (PSO) [34], and other algorithms [4, 35, 36]. Jarboui et al. [37] put forward a combinatorial particle swarm optimization for solving MRCPSP; the computational analyses in Zhang et al. [34] showed that the PSO-based approach for the RCPSP was more efficient than the GA approach due to its features, such as the one-way experience sharing mechanism during the PSO search. Existing publications indicate that the PSO method has comparable or even superior performance when solving many NP-hard problems and has a fast and stable convergence [38], so, particle swarm optimization, inspired by the social behaviors of animals like fish schooling and bird flocking and proposed for optimization [39], is adopted in this study to develop a combinatorial-priority-based hybrid PSO (CP-based HPSO) algorithm to solve the MRCMPSP, where the combinatorial PSO and priority-based PSO are designed to assign modes to activities and to schedule activities, respectively.

In brief, the following techniques are used in this paper. First, the scheduling problem in project management takes multiple modes for each activity, multiple project scheduling, and hybrid uncertainty environment into consideration comprehensively. Second, as the iron triangle inextricably linked with measuring the success of project management, cost, time, and quality are proposed as the optimal control criteria in this paper. So, a time/cost/quality trade-off optimal control model is established to solve the MRCMPSP under a fuzzy random environment in project management. Another technique used here is the CP-based HPSO, which was developed based on the particular nature of the MRCMPSP and the standard PSO. The final contribution of this paper is to put forward a practical application. A large scale hydropower construction project in the southwest region of China is used to illustrate the maneuverability, scientific advanced nature, and the effectiveness of the proposed model and optimization method.

The remainder of this paper is organized as follows: Section 2 describes the key problem statement for the MRCMPSP. A multiobjective optimization model for MRCMPSP under a fuzzy random environment is then formulated in Section 3. In Section 4, a multiobjective CP-based HPSO algorithm is proposed to solve the model. In Section 5, a practical case is used to demonstrate the practicality of the modeling method and the efficacy of the developed algorithm. Finally, concluding remarks are given in Section 6.

## 2. Key Problem Statement

### 2.1. Problem Description

**Figure 1:**
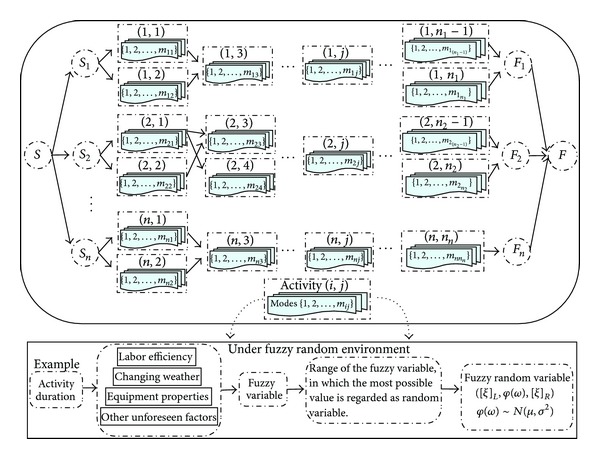
Problem description for MRCMPSP.

As the key problem in project management, project scheduling exists in all kinds of reality problems. It represents the conversion of project goals into an achievable methodology for their completion; it creates a timetable and reveals the network logic that relates project activities to each other in a coherent fashion. With more and more complex project management issues, RCPSP and MRCPSP have been proposed and well applied in terms of a single project. The project scheduling problem considered in this paper is from a large scale hydropower construction project in the southwest region of China, in which the main project comprises three parallel projects (i.e., a river diversion construction project, a river dam construction project, and a water power generation system construction project) which have no impact on each other, but each project has many activities with precedence relationships and shared resources. With these issues in mind, this paper focuses on a multimode resource-constrained multiple project scheduling problem (MRCMPSP), which contains mode selection problem and activities scheduling problem for multiple projects. The MRCMPSP considered adheres to the following assumptions, the details of which are in Figure 1.

1. The construction project considered in this paper consists of *n* parallel projects, and two dummy projects (i.e., *S* and *F*) are introduced to denote the start and the completion of the construction project so are not allocated any costs or processing time. Similarly, each project (i.e., *i* ∈ *N* = {1,2,…, *n*}) consists of *n*_*i*_ activities (i.e., *j* ∈ *N*_*i*_ = {1,2,…, *n*_*i*_}) and two dummy activities (i.e., *S*_*i*_ and *F*_*i*_) which represent the initial and final activities.
2. Precedence relationships are the finish-start with a time lag of zero, which means that each activity (i.e., (*i*, *j*) ∈ *A*) can be started if and only if all of its predecessors (i.e., *P*_*ij*_) have been completed. There is no additional time allocated to activity switching. In addition, when an activity begins, it cannot be interrupted.
3. Each activity (*i*, *j*) must be performed in only one of *m*_*ij*_ possible modes, with each activity mode (i.e., *m* ∈ *M*_*ij*_ = {1,2,…, *m*_*ij*_}) possibly having different processing times, different resource requirements, and different quality indexes. Mode switching is not allowed when an activity is being executed.
4. The shared resources that activities require can be divided into two types, renewable resources (i.e., *r* ∈ *R*) which are limited period by period, such as manpower and equipment, and nonrenewable resources (i.e., *k* ∈ *K*) which are limited for the entire project, such as building materials.
5. The interrelationship among activities is executed in a certain order using an activity-on-node (AON) representation, in which the node represents an activity and path arrows demonstrate the logical sequencing from node to node through the network.
6. The starting time of each project is dependent upon the project's priorities and the characteristics of the first activity set in each project and is also dependent upon an unlimited number of other reasons.

### 2.2. Motivation for Employing Fuzzy Random Variables in the MRCMPSP

The need to address uncertainty in project management is widely recognized, as uncertainties exist in a variety of system components. As a result, the inherent complexity and stochastic uncertainty existing in real-world RCPSP decision making processes have essentially placed them beyond conventional deterministic optimization methods. Here is an example to illustrate the uncertainty.

*Activity Duration*. With the complexity of uncertainty factors, such as the changing weather, equipment properties, labor efficiency, materials supply, and coordination problems among stakeholders, activity duration is a typical uncertain variable. Van de Vonder et al. [11], Herroelen and Leus [12], and Bidot et al. [13] considered the project scheduling with stochastic activity durations, Choi et al. [14] proposed a novel way of addressing the uncertainties in durations and costs. However, sometimes random variables cannot adequately describe activity durations because probability distributions for some activity durations may be unknown or just partially known due to the lack of statistical data. Pinto [40] proposed that there are a number of alternative ways to estimate durations in project management, including past experience, expert opinion, and mathematical derivation, and the typical expression is within the most optimistic, the most likely, and the most pessimistic duration estimates for each activity, which gives rise to the fuzzy set theory to describe the uncertainty of activity duration. Subsequently, Xu et al. [33] used fuzzy number to denote activity duration and Xu and Zhang [28] used fuzzy random variable to denote the due date of the project. With the complexity of uncertainty factors and uniqueness of MRCMPSP in a new large scale construction project, activity duration is flexible or imprecise in nature, data of which were collected from different experienced engineers (i.e., *q* = 1,2,…, *E*, where *q* is the index of engineers), with each being an interval (i.e., [*l*_*q*_, *r*_*q*_]) with the highest possible value (i.e., *m*_*q*_), such that “the duration of upstream cofferdam is between 1360 and 1570 hour, and the most possible value is 1450 hour”. Since different engineers have different views on activity duration, it is necessary to first determine the minimum duration (i.e., [*ξ*]_*L*_) and the maximum duration (i.e., [*ξ*]_*R*_) of all collected data, respectively. Then the maximum likelihood method is used to deal with all the most possible values and to find whether they approximately follow a normal distribution (i.e., *N*(*μ*, *η* ^2^)), which is derived by that the most possible value of the activity duration can be represented using a normal distribution [40]. Therefore the duration of each activity is characterized as a triangular fuzzy random variable (i.e., ([*ξ*]_*L*_, *φ*(*ω*), [*ξ*]_*R*_), where *φ*(*ω*) ~ *N*(*μ*, *η* ^2^)).

Similarly, due to a lack of determined data, environmental variations, engineering technology advancements, engineer's different experiences, and other unforeseen factors, fuzzy random variables are employed to describe the uncertainty of unit costs, quality indexes, and the resources required [28] in the MRCMPSP. Therefore, there is a strong motivation to use a fuzzy random environment for the MRCMPSP discussed in this paper.

### 2.3. Description for the MRCMPSP under a Fuzzy Random Environment

Project scheduling problem in large scale construction projects is a crucial task and must be dealt with urgently because of high costs, long project duration, and other important reasons; the MRCMPSP in this paper consists in scheduling activities and activity-mode combinations under the presence of precedence and resource constraints. With the complexity of uncertainty factors and uniqueness of MRCMPSP in a new large scale construction project, it is very suitable and necessary to use fuzzy random environment to describe the hybrid uncertain construction environment. For instance, when each activity is scheduled and executed in one mode, renewable resources and nonrenewable resources are required, the quantity of each is a fuzzy random variable; each mode for one activity possibly needs different quantity of resources. As the most typical goals in project management, project time, cost, and quality have received significant attention [41, 42] and should be taken into account synchronously when solving the MRCMPSP in this paper. Thus, the purpose of this paper is to ensure the completion of a large scale construction project with minimum possible project time and cost and a maximum possible project quality when solving the MRCMPSP under a fuzzy random environment. This leads to the following modelling.

## 3. Modelling

The MRCMPSP discussed in this paper consists in scheduling project activities and activity-mode combinations to achieve objectives under the presence of precedence and resource constraints. In this section, a time/cost/quality trade-off (TCQT) optimization model for the MRCMPSP under a fuzzy random environment is constructed, and its corresponding mathematical description is presented as follows using the following notation.

*Index and Sets*

 
*i*: Project index, *i* = 1,2, ,…, *n* ∈ *N*
 
*j*: Activity index in project *i*, *j* = 1,2,…, *n*_*i*_ ∈ *N*_*i*_
 (*i*, *j*): Activity index for all projects, (*i*, *j*) ∈ *A*
 
*k*: Nonrenewable resource type index, *k* ∈ *K*
 
*r*: Renewable resources type index, *r* ∈ *R*
 
*m*: Mode index, *m* = 1,2,…, *m*_*ij*_ ∈ *M*_*ij*_ (is the number of possible modes of activity (*i*, *j*))
 
*t*: Period index
 
*I*_*t*_: Set of all ongoing activities at period *t*
 
*P*_*ij*_: Set of all predecessors in project *i* of activity (*i*, *j*), *v* ∈ *P*_*ij*_.

*Certain Parameters*

 
*C*_*ij*_: Fixed cost of activity (*i*, *j*) with normal duration, (*i*, *j*) ∈ *A*
 
*T*: The upper bound of the project completion time, *t* ∈ {1,2,…, *T*}
 
*B*: Available total budget
 
*R*_*k*_^*υ*^: Total amount of available nonrenewable resource *k*, *k* ∈ *K*
 
*R*_*rt*_^*ρ*^: Capacity of renewable resource *k* available at period *t*, *r* ∈ *R*, *t* ∈ {1,2,…, *T*}
 
*EV*_*ij*_: Earned value of activity (*i*, *j*), (*i*, *j*) ∈ *A*
 
*w*_*i*_: Weight of project *i* compared to other projects in the whole project.

*Uncertain Parameters*

 
${\tilde{\overset{-}{d}}}_{ij}$: Normal duration of activity (*i*, *j*), (*i*, *j*) ∈ *A*
 
${\tilde{\overset{-}{d}}}_{ij}^{m}$: Crashed duration of activity (*i*, *j*) executed in mode *m*, (*i*, *j*) ∈ *A*, *m* ∈ *M*_*ij*_
 
${\tilde{\overset{-}{c}}}_{ij}$: Unit variable cost of activity (*i*, *j*), (*i*, *j*) ∈ *A*
 
${\tilde{\overset{-}{k}}}_{ij}$: Unit crashing cost of activity (*i*, *j*), (*i*, *j*) ∈ *A*
 
${\tilde{\overset{-}{q}}}_{ij}^{m}$: Quality index of activity (*i*, *j*) executed in mode *m*, (*i*, *j*) ∈ *A*, *m* ∈ *M*_*ij*_
 
${\tilde{\overset{-}{r}}}_{ijk}^{m}$: Units of nonrenewable resource *k* required by activity (*i*, *j*) executed in mode *m*, (*i*, *j*) ∈ *A*, *k* ∈ *K*, *m* ∈ *M*_*ij*_
 
${\tilde{\overset{-}{r}}}_{ijr}^{m}$: Per period usage of renewable resource *r* required by activity (*i*, *j*) executed in mode *m*, (*i*, *j*) ∈ *A*, *r* ∈ *R*, *m* ∈ *M*_*ij*_.

*Variables*

 
*S*_*ij*_: Start time of activity (*i*, *j*), (*i*, *j*) ∈ *A*
 
*F*_*ij*_: Finish time of activity (*i*, *j*), (*i*, *j*) ∈ *A*
 
$x_{ij}^{m}=\{\begin{array}{cc} 1, & \text{if}\;\text{activity}\;\left(i,j\right)\;\text{is}\;\text{being}\;\text{executed}\;\text{in}\;\text{mode}\;m,\\ 0, & \text{otherwise}, \end{array}$
 
$y_{ijt}^{m}=\{\begin{array}{cc} 1, & \text{if}\;\text{activity}\;\left(i,j\right)\;\text{in}\;\text{mode}\;m\;\text{is}\;\text{scheduled}\;\text{at}\;\text{time}\;t,\\ 0, & \text{otherwise}. \end{array}$

### 3.1. Dealing with Fuzzy Random Variables

**Figure 2:**
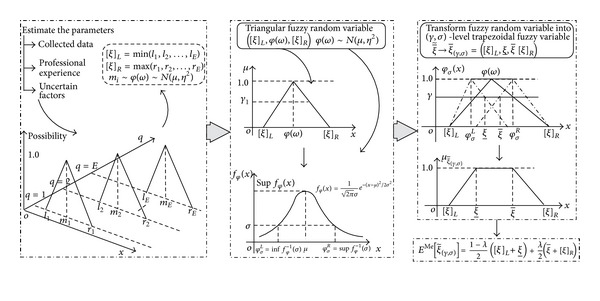
Transformation method of the fuzzy random parameters.

The fuzzy random variables in this study ensure a greater data accuracy but make the MRCMPSP significantly more difficult to solve. One strategy is to employ a transformation method to convert the fuzzy random variables into real numbers; thus, the hybrid crisp approach put forward by Xu et al. [43] first transforms the fuzzy random parameters into (*γ*, *σ*)-level trapezoidal fuzzy variables, which are subsequently defuzzified using an expected value operator with an optimistic-pessimistic index. Without a loss of generality, denote the fuzzy random variables as $\tilde{\overset{-}{\xi }}=({\left[\xi \right]}_{L},\varphi (\omega ),{\left[\xi \right]}_{R})$; here *φ*(*ω*) ~ *N*(*μ*, *η* ^2^) with a probability density function *f*_*φ*_(*x*). The procedure is summarized as follows and the transformation process is illustrated in Figure 2.

(1) Estimate the parameters [*ξ*]_*L*_, [*ξ*]_*R*_, *μ*, and *η* ^2^ from the collected data and professional experience using statistical methods. Specifically, the minimum value of all *l*_*q*_ and the maximal value of all *r*_*q*_ for each parameter in the survey data were selected as [*ξ*]_*L*_ and [*ξ*]_*R*_, respectively. *μ* and *η* ^2^ can be estimated using the maximum likelihood method and justified by a chi-square goodness-of-fit test.
(2) Obtain the possibility level of the fuzzy variable (i.e., *γ*) and the probability level of the random variable (i.e., *σ*), where *r* ∈ [([*ξ*]_*R*_ − [*ξ*]_*L*_)/([*ξ*]_*R*_ − [*ξ*]_*L*_ + *φ*_*σ*_^*R*^ − *φ*_*σ*_^*L*^), 1], *σ* ∈ [0, sup*f*_*φ*_(*x*)].
(3) Let *φ*_*σ*_ be the *σ*-cut of the random variable *φ*(*ω*); that is, *φ*_*σ*_ = [*φ*_*σ*_^*L*^, *φ*_*σ*_^*R*^] = {*x* ∈ *R* | *f*_*φ*_(*x*) ≥ *σ*}, where $\varphi_{\sigma }^{L}=\inf\{x\in R|f_{\varphi }(x)\geq \sigma \}=\mu -\sqrt{-2\eta^{2}\ln(\sqrt{2\pi }\sigma \eta )}$ and *φ*_*σ*_^*R*^ = sup{*x* ∈ *R* | *f*_*φ*_(*x*)≥*σ*} = $\mu +\sqrt{-2\eta^{2}\ln(\sqrt{2\pi }\sigma \eta )}$.
(4) Transform the fuzzy random variable into the (*γ*, *σ*)-level trapezoidal fuzzy variable ${\tilde{\overset{-}{\xi }}}_{\left(\gamma ,\sigma \right)}$ by equation $\tilde{\overset{-}{\xi }}\to {\tilde{\xi }}_{\left(\gamma ,\sigma \right)}=({\left[\xi \right]}_{L},\underline{\xi },\bar{\xi },{\left[\xi \right]}_{R})$, where $\underline{\xi }={\left[\xi \right]}_{R}-\gamma ({\left[\xi \right]}_{R}-\varphi_{\sigma }^{L})$ and $\bar{\xi }={\left[\xi \right]}_{L}+\gamma (\varphi_{\sigma }^{R}-{\left[\xi \right]}_{L})$.
(5) Defuzzify the (*γ*, *σ*)-level trapezoidal fuzzy variables using an expected value operator with an optimistic-pessimistic index *λ* as follows:
  (1) $$\begin{array}{c} E^{Me}\left[{\tilde{\xi }}_{\left(\gamma ,\sigma \right)}\right]=\frac{\left(1-\lambda \right)}{2}\left({\left[\xi \right]}_{L}+\underline{\xi }\right)+\frac{\lambda }{2}\left(\bar{\xi }+{\left[\xi \right]}_{R}\right). \end{array}$$

In this paper, all probability levels and possibility levels are denoted as *σ* and *γ*, respectively.

### 3.2. TCQT Optimization Model Objective Functions for the MRCMPSP

*Weighted Project Makespan*. The activities in project *i* are well organized and numbered 0 to *n*_*i*_ + 1, where the 0th and (*n*_*i*_ + 1)th activities are dummy activities representing the start and end activities, respectively; then *S*_*i*,0_ and *F*_*i*,*n*_*i*_+1_ are used to represent the start and finish times of project *i*, respectively, so the duration of project *i* is (*F*_*i*,*n*_*i*_+1_ − *S*_*i*,0_) based on previous studies like [34]. For the whole project, the duration is the makespan between the project start time and project completion time, which means that it is the maximum value of all the finish times of all the independent projects, which can be expressed as max_*i*_ *F*_*i*,*n*_*i*_+1_.

In a large scale project which contains many projects, the whole project duration is not able to adequately describe the characteristics of the whole project and each project. Considering the different importance of each project and the different requirements of both the whole project duration and the single project duration, it is strongly recommended to mark the objective function of the weighted project makespan. Let *z*_1_ be the weighted project makespan, which can be expressed as follows:

(2) $$\begin{array}{c} z_{1}=\lambda_{1}\times \underset{i}{\max}\;F_{i,n_{i}+1}+\lambda_{2}\times \sum_{i=1}^{n}\left(\omega_{i}\times \left(F_{i,n_{i}+1}-S_{i,0}\right)\right), \end{array}$$

where *λ*_1_ and *λ*_2_ represent the weight of project duration and the weight of single project duration time, respectively, and *λ*_1_ + *λ*_2_ = 1, ∑_*i*=1_^*n*^ *ω*_*i*_ = 1.

*Project Cost*. Project cost optimization is a crucial consideration in large scale project management and must be dealt with urgently because of the high expenses, the impact on project quality and durations, and so forth. Usually total project cost changes occur because of changes to fixed costs, unit costs, duration, the mode activity, and so forth. Therefore, project managers aim to achieve the best option for the execution of the process by minimizing the total project cost. In review of the previous studies [33, 44], it can be derived that the total project cost for the MRCMPSP in this paper is composed of fixed cost (i.e., *C*_*ij*_), variable cost (i.e., ${\tilde{\overset{-}{c}}}_{ij}\times {\tilde{\overset{-}{d}}}_{ij}^{m}$), and the crashing cost (i.e., ${\tilde{\overset{-}{k}}}_{ij}\times ({\tilde{\overset{-}{d}}}_{ij}-{\tilde{\overset{-}{d}}}_{ij}^{m})$) of each activity. Here, the fuzzy random variables (i.e., ${\tilde{\overset{-}{c}}}_{ij}$, ${\tilde{\overset{-}{k}}}_{ij}$, ${\tilde{\overset{-}{d}}}_{ij}^{m}$, and ${\tilde{\overset{-}{d}}}_{ij}$) are converted into real numbers (i.e., $E^{Me}[{\tilde{c}}_{ij(\gamma ,\sigma )}]$, $E^{Me}[{\tilde{k}}_{ij(\gamma ,\sigma )}]$, $E^{Me}[{\tilde{d}}_{ij(\gamma ,\sigma )}^{m}]$, and $E^{Me}[{\tilde{d}}_{ij(\gamma ,\sigma )}]$) using the above approach. Let *z*_2_ be the total project cost, which can be expressed as follows:

(3) z2=∑i=1n∑j=1ni∑m=1mij(Cij+EMe[c~ij(γ,σ)]EMe[d~ij(γ,σ)m]xijm+EMe[k~ij(γ,σ)]EMe[d~ij(γ,σ)−d~ij(γ,σ)m]xijm).

*Project Quality*. Besides consideration of the resource constraints, project duration, and project management cost, project quality is another important objective to optimize. In order to realize the quantization of the quality index, let *EQV*_*ij*_ express the earned quality value of activity (*i*, *j*), which can be obtained using the following formula: $EQV_{ij}=\sum_{m=1}^{m_{ij}}EV_{ij}\times {\tilde{\overset{-}{q}}}_{ij}^{m}x_{ij}^{m}$. Considering the differing importance of each project and the different requirements of each project, a weighted project quality is strongly recommended to mark the total quality of the whole project. Here, the fuzzy random variable (i.e., ${\tilde{\overset{-}{q}}}_{ij}^{m}$) is converted into a real number (i.e., $E^{Me}[{\tilde{q}}_{ij(\gamma ,\sigma )}]$) using the above approach. Let *z*_3_ be the weighted project quality, which can be expressed as follows:

(4) z3=∑i=1nωi1∑j=1niEVij×∑j=1niEQVij=∑i=1nωi1∑j=1niEVij×∑j=1ni∑m=1mijEVij×EMe[q~ij(γ,σ)m]xijm.

### 3.3. TCQT Optimization Model Constraints for MRCMPSP

*Mode Uniqueness Constraint*. Each activity must be performed in only one mode, and mode switching is not allowed when an activity is being executed, which can be ensured using the following constraint set:

(5) $$\begin{array}{c} \sum_{m=1}^{m_{ij}}x_{ij}^{m}=1,\;\forall \left(i,j\right)\in A. \end{array}$$

*Budget and Completion Time Constraints*. In a large scale project, it is extremely important to draw up the construction contract with deterministic limits for total budget and project duration before any activities are executed; then the following constraints are made:

(6) $$\begin{array}{c} z_{2}\leq B,\\ \underset{i}{\max}\;F_{i,n_{i}+1}\leq T,\;\forall i\in N,\;\;n_{i}\in N_{i}. \end{array}$$

*Time Constraints*. Constraint sets (7) compute the start time for each activity, which is obtained from the decision variables and the mode selection, where *M* is an infinite number which ensures that the start time of each activity is no greater than the time for which it is scheduled. Each activity (*i*, *j*) is executed only once, and the total number of periods that it is executed in is equal to its duration when executed in mode *m*. In addition, its corresponding finish time *F*_*ij*_ can be obtained using (9). Here, the fuzzy random variable ${\tilde{\overset{-}{d}}}_{ij}^{m}$ is converted into a real number (i.e., $E^{Me}[{\tilde{d}}_{ij\left(\gamma ,\sigma \right)}^{m}]$) using the above approach. With these in mind, the corresponding constraints are listed as follows:

(7) $$\begin{array}{c} y_{ijt}^{m}\times t+M\left(1-y_{ijt}^{m}\right)\geq S_{ij},\;\forall \left(i,j\right)\in A,\;\;m\in M_{ij}, \end{array}$$

(8) $$\begin{array}{c} \sum_{t=0}^{T}y_{ijt}^{m}=E^{Me}\left[{\tilde{d}}_{ij\left(\gamma ,\sigma \right)}^{m}\right]\times x_{ij}^{m},\;\forall \left(i,j\right)\in A,\;\;m\in M_{ij}, \end{array}$$

(9) $$\begin{array}{c} F_{ij}=S_{ij}+\sum_{m=1}^{m_{ij}}E^{Me}\left[{\tilde{d}}_{ij\left(\gamma ,\sigma \right)}^{m}\right]x_{ij}^{m},\;\forall \left(i,j\right)\in A. \end{array}$$

*Precedence Constraint*. In a project, precedence is an important basic term ensuring arrangement rationality. With this in mind, and from the assumptions in the key problem statement, an activity can be started if and only if all its predecessors have already been completed. It is important that none of the precedence constraints are violated for all predecessors of activity (*i*, *j*) as shown in the following:

(10) $$\begin{array}{c} F_{iv}\leq S_{ij},\;\forall v\in P_{ij},\;\;\left(i,j\right)\in A. \end{array}$$

*Resource Constraints*. Renewable resources and nonrenewable resources are the two types of resource activities required. Constraint set (11) forces the total nonrenewable resource units utilized to be no greater than the total nonrenewable resources available, whereas constraint set (12) forces the total renewable resource units utilized in every period to be no greater than the available renewable resources for any period. Here, the fuzzy random variables ${\tilde{\overset{-}{r}}}_{ijk}^{m}$ and ${\tilde{\overset{-}{r}}}_{ijr}^{m}$ are converted into real numbers (i.e., *E* ^*Me*^[*r*_*ijk*(*γ*,*σ*)_^*m*^] and *E* ^*Me*^[*r*_*ijr*(*γ*,*σ*)_^*m*^]). Consider

(11) ∑i=1n∑j=1ni∑m=1mijEMe[rijk(γ,σ)m]xijm≤Rkυ,  ∀(i,j)∈A,  m∈Mij,  k∈K,

(12) ∑i=1n∑j=1ni∑m=1mijEMe[rijr(γ,σ)m]yijtm≤Rrtρ,  ∀(i,j)∈It,  m∈Mij,  r∈R.

*Logical Constraints*. In order to describe the nonnegative variables and the 0 − 1 variables in the model for a practical situation, the following constraints are presented:

(13) $$\begin{array}{c} S_{ij}\geq 0,\;F_{ij}>0,\;\forall \left(i,j\right)\in A,\;\;m\in M_{ij},\\ x_{ij}^{m}=0\;\text{or}\;1,\;y_{ijt}^{m}=0\;\text{or}\;1,\;\forall \left(i,j\right)\in A,\;\;m\in M_{ij}. \end{array}$$

### 3.4. Model Formulation

In project management, RCPSP is committed to a schedule of activities to minimize project duration. As a novel extension of the RCPSP, the MRCPSP has emerged and has been verified. This allows for the diversity in activity modes that exists in reality, where each activity must be executed in only one mode which represents a relationship between the resource requirements of the activity and its duration. Based on the RCPSP and the MRCPSP, multimode resource-constrained multiple project scheduling problem (MRCMPSP) is proposed first in this paper, which takes both multiple activity modes and multiple parallel projects scheduling into consideration. In this optimization model, duration, cost, and quality, as the most typical goals in project management, are comprehensively and systematically analyzed and the trade-off is optimized, which improves the overall construction project efficiency. In this study, fuzzy random uncertainty is adopted to describe the hybrid uncertain environment for MRCMPSP, which ensures greater data accuracy. A hybrid crisp approach is used to transform the fuzzy random parameters into (*γ*, *σ*)-level trapezoidal fuzzy variables, which are subsequently defuzzified using an expected value operator with an optimistic-pessimistic index as shown in (1). With these in mind, an expected value model for weighted makespan/cost/quality trade-off optimization for the MRCMPSP under a fuzzy random environment is established, which aims to schedule activities and assign activity modes to achieve the objectives under the presence of precedence and resource constraints. From the notations, objective functions, and constraints outlined above, the multiobjective expected value model for the MRCMPSP can be formulated in the following:

(14) min z1=λ1×maxi Fi,ni+1+λ2×∑i=1n(ωi×(Fi,ni+1−Si,0))‍,min z2=∑i=1n∑j=1ni∑m=1mij(Cij+EMe[c~ij(γ,σ)]EMe×[d~ij(γ,σ)m]xijm+EMe[k~ij(γ,σ)]EMe×[d~ij(γ,σ)−d~ij(γ,σ)m]xijm),max z3=∑i=1nωi1∑j=1niEVij×∑j=1ni∑m=1mijEVij×EMe[q~ij(γ,σ)m]xijms.t. λ1+λ2=1,∑i=1nwi=1, i∈N,∑m=1mijxijm=1, ∀(i,j)∈A,z2≤B,maxi Fi,ni+1≤T, ∀i∈N,  ni∈Ni,Fij=Sij+∑m=1mijEMe[d~ij(γ,σ)m]xijm,∀(i,j)∈A,yijtm×t+M(1−yijtm)≥Sij,∀(i,j)∈A,  m∈Mij,∑t=0Tyijtm=EMe[d~ij(γ,σ)m]×xijm,∀(i,j)∈A,  m∈Mij,Fiv≤Sij, ∀v∈Pij,  (i,j)∈A,∑i=1n∑j=1ni∑m=1mijEMe[rijk(γ,σ)m]xijm≤Rkυ,∀(i,j)∈A,  m∈Mij,  k∈K,∑i=1n∑j=1ni∑m=1mijEMe[rijr(γ,σ)m]yijtm≤Rrtρ,∀(i,j)∈It,  m∈Mij,  r∈R,t∈{1,2,…,T},xijm=0  or  1, yijtm=0  or  1,∀(i,j)∈A,  m∈Mij,Sij≥0, Fij>0, ∀(i,j)∈A,  m∈Mij.

## 4. Combinatorial-Priority-Based Hybrid PSO Algorithm for Solving the MRCMPSP

As a generalization of the classical project scheduling problem, MRCPSP belongs to the class of NP-hard optimization problems. As shown by Potts and Kovalyov [30], Detti [31], and Pinedo [45], exact methods are unable to find optimal solutions for MRCPSP. In this case, several heuristic procedures have been proposed to solve MRCPSP, such as genetic algorithms as shown in [46, 47], simulated annealing algorithm [3], particle swarm optimization [37], and local search procedure [48]. Since many kinds of PSO have been tested and verified for solving the RCPSP and MRCPSP, furthermore, based on the particular nature of our model and the easy-to-implement software development of PSO algorithm, the PSO is adopted in this study to develop a combinatorial-priority-based hybrid PSO (CP-based HPSO) algorithm for solving the MRCMPSP.

Particle swarm optimization is a population-based self-adaptive search stochastic optimization technique proposed by Kennedy and Eberhart [39], which was inspired by the social behavior of animals such as fish schooling and birds flocking to find a promising position for certain objectives in a multidimensional space [38, 49]. Similar to the evolutionary computation technique, the PSO maintains a population of particles, where each particle represents a potential solution to an optimization problem. The PSO formula is shown below:

(15) Vl(τ+1)=w(τ)Vl(τ)+cprp(Pl−Xl(τ)) +cgrg(G−Xl(τ)),

(16) $$\begin{array}{c} X_{l}\left(\tau +1\right)=V_{l}\left(\tau +1\right)+X_{l}\left(\tau \right), \end{array}$$

(17) $$\begin{array}{c} w\left(\tau \right)=w\left(T\right)+\frac{\tau -T}{1-T}\left[w\left(1\right)-w\left(T\right)\right], \end{array}$$

where *l* = 1,2,…, *L* (population size); *τ* = 1,2,…, *T* (iteration limit); *X*_*l*_(*τ*) = (*x*_*l*1_(*τ*), *x*_*l*2_(*τ*),…, *x*_*lH*_(*τ*)), and *V*_*l*_(*τ*) = (*v*_*l*1_(*τ*), *v*_*l*2_(*τ*),…, *v*_*lH*_(*τ*)) denote the *H*-dimension (problem dimension) position and velocity for the *l*th particle in the *τ*th iteration, respectively; *P*_*l*_ = (*p*_*l*1_, *p*_*l*2_,…, *p*_*lH*_) and *G* = (*G*_1_, *G*_2_,…, *G*_*H*_) denote the personal best position of the *l*th particle encountered after *τ* iterations and global best, respectively; *c*_*p*_ and *c*_*g*_ are the acceleration constants and *r*_*p*_ and *r*_*g*_ are random real numbers drawn from *U*(0,1); *w*(*τ*), the inertia weight used to determine the influence of the previous velocity on the new velocity. Equation (15) is used to calculate the particle's new velocity, (16) is used to update the particle moving toward a new position [50], and (17) shows how the adaptive inertia weights vary with iterations [50].

### 4.1. Weight-Sum Procedure for Dealing with the Multiobjective Factor

Based on the natural characteristics of the mathematical model in (14), the aggregating approach with weighted-sum form is used to deal with the multiobjective factor in this paper. Only when the solution set is convex [51] can the aggregated objective in the weighted-sum form be used to find the optimal Pareto solutions, and the convexity of the above mathematical model and its solution set can be easily proved. So, in this paper, the weight-sum procedure is adopted and the estimated maximal value is used to divide the dimensions and unify the orders of magnitude in the three objectives [33]. The basic procedure is as follows:

(1) estimate the maximal values *z*_1_^max^, *z*_2_^max^, and *z*_3_^max^ of *z*_1_, *z*_2_, and *z*_3_, respectively;
(2) calculate and standardize the *z*_1_′, *z*_2_′, and *z*_3_′ as follows:
  (18) $$\begin{array}{c} z_{1}^{\prime }=\frac{z_{1}}{z_{1}^{\max}},\;\;z_{2}^{\prime }=\frac{z_{2}}{z_{2}^{\max}},\;\;z_{3}^{\prime }=\frac{z_{3}}{z_{3}^{\max}}, \end{array}$$
(3) the weighted-sum objective function *z* = min(*η*_1_ *z*_1_′ + *η*_2_ *z*_2_′ − *η*_3_ *z*_3_′), where *η*_1_ + *η*_2_ + *η*_3_ = 1.

The weights *η*_1_, *η*_2_, and *η*_3_ are proposed for the weighted project makespan, project cost, and project quality, respectively, all of which have been provided by the decision makers and reflect the importance of each objective from their view. For a given individual, the fitness value function is expressed as follows:

(19) $$\begin{array}{c} \text{Fitness}\left(X_{l}\left(\tau \right)\right)=\eta_{1}z_{1}^{\prime }+\eta_{2}z_{2}^{\prime }+\eta_{3}z_{3}^{\prime }. \end{array}$$

### 4.2. Encoding Scheme and Decoding Scheme for CP-Based HPSO

In PSO, the position of a particle is indicated by a vector which presents the solution of the investigated problem. According to the nature of CP-based HPSO proposed in this paper, the execution modes and the start time of all activities are considered the problem dimensions (i.e., 2∑_*i*=1_^*N*^ *N*_*i*_), and other variables are treated as hidden parameters. In this CP-based HPSO, let *X*_*l*_^1^(*τ*) denote the execution modes; *X*_*l*_^2^(*τ*) denote the activity priorities which represent the activity start times, while the placement of which reflectively corresponds to the activity indexes. Therefore, let *X*_*l*_(*τ*) = [*X*_*l*_^1^(*τ*), *X*_*l*_^2^(*τ*)] and *V*_*l*_(*τ*) = [*V*_*l*_^1^(*τ*), *V*_*l*_^2^(*τ*)] denote the 2*H*-dimension position and velocity for the *l*th particle in the *τ*th iteration, respectively. Similarly, let *P*_*l*_ = [*P*_*l*_^1^, *P*_*l*_^2^] and *G* = [*G* ^1^, *G* ^2^] denote the 2*H*-dimension personal best position and global best position, respectively, in which *X*_*l*_^1^(*τ*), *V*_*l*_^1^(*τ*), *P*_*l*_^1^(*τ*), and *G* ^1^(*τ*) are the representation in the combinatorial PSO; *X*_*l*_^2^(*τ*), *V*_*l*_^2^(*τ*), *P*_*l*_^2^(*τ*), and *G* ^2^(*τ*) are the representation in the priority-based PSO.

With known activity priorities, there are two types of schedule generation schemes (SGS, i.e., serial schedule generation scheme (SSGS) and parallel schedule generation scheme (PSGS)) usually used for generating the RCPSP schedule [52]. Where SSGS can cause a larger deviation in the optimization results with inappropriate priority rules, the PSGS can decrease this but has a longer scheduling time. Further, when focusing on multiple projects, a hybrid schedule generation scheme (HSGS) with a combination of SSGS and PSGS is proposed in this study. The HSGS has many stages and each stage is made up as shown in the right part of Figure 4, wherein *F*_*n*_ is the set of activities which have been completed at stage *n*; *A*_*n*_ is the set of activities which are ongoing and have not yet been completed at stage *n*; *E*_*n*_ is the set of activities which are going to be processed and whose precedence activities have already been completed or are still in *E*_*n*_; and *U*_*n*_ is the set of all remaining activities at stage *n*.

### 4.3. Overall Procedure of CP-Based HPSO

As we can see, the MRCMPSP consists of two different subproblems: the assignment of modes to activities and the scheduling of activities to achieve the objectives. Aimed at the first problem, a combinatorial PSO proposed by Jarboui et al. [37] is referenced while a priority-based PSO is proposed to deal with the scheduling problem. To solve the MRCMPSP, in accordance with the standard PSO, the combinatorial PSO is used to initialize and update position *X*_*l*_^1^(*τ*) and velocity *V*_*l*_^1^(*τ*) (*l* = 1,2,…, *L*) in a novel method. The advantage of the priority-based PSO is the priority representation for position *X*_*l*_^2^(*τ*) and a hybrid schedule generation scheme to generate the MRCMPSP schedule with known activity priorities. Based on the basic PSO, and utilizing the innovation and highlights of the combinatorial PSO and priority-based PSO, the overall procedure to implement the CP-based HPSO for the MRCMPSP is expounded as follows.

#### 4.3.1. Initialization for CP-Based HPSO

**Step 1 (initialize particles)**

Initialize *L* particles as a swarm; set iteration *τ* = 0. For *l* = 1,2,…, *L*, generate the *H*-dimension position of particle *l* with an integer vector *X*_*l*_^1^(0), where the value of *x*_*lh*_^1^(0) is randomly selected from (1,2,…, *m*_*h*_) (combinatorial PSO); generate the *H*-dimension random position *X*_*l*_^2^(0) within [0,1] (priority-based PSO); generate 2*H*-dimension random velocity *V*_*l*_(0) within [−1,1].

**Step 2**

Check the feasibility.  

*Step 2* *.1*. For *l* = 1,2,…, *L*, decode the particles to solutions; if the feasibility criterion is met by all particles, then the particles are feasible; go to Step 3. Otherwise, go to Step 2.2.

*Step 2.2*. It is necessary to check and adjust solutions to avoid nonrenewable resource infeasibility. For the infeasible nonrenewable resources, select an activity (*i*, *j*) with multiple execution modes.

*Step 2.3*. Select a new mode *m*′ within 1,2,…, *m*_*ij*_ for activity (*i*, *j*) randomly. Then check whether (11) is met.

*Step 2.4*. If (11) is met, replace *m*′ with *m* and then go to Step 3. Otherwise, repeat Step 2.3 until all modes of activity (*i*, *j*) have been iterated.

**Step 3 (calculate the initial personal best and global best)**

For *l* = 1,2,…, *L*, compute the fitness value of each particle *l* based on (19) and identify the personal best of each particle and the global best in the swarm, where *P*_*l*_^1^(0) = *X*_*l*_^1^(0) and *P*_*l*_^2^(0) = *X*_*l*_^2^(0). Then proceed to the next iteration 1.

#### 4.3.2. Updating and Schematic Procedure for CP-Based HPSO

**Step 1 (velocity and position updating)**

For each particle *l* in the particle swarm, the updating mechanism proposed in the combinatorial PSO [37] is used to update the velocity *V*_*l*_^1^(*τ* − 1) and position *X*_*l*_^1^(*τ* − 1). At the same time, update and adjust position *X*_*l*_^2^(*τ* − 1) and velocity *V*_*l*_^2^(*τ* − 1) of the *l*th particle using the priority-based PSO (i.e., (15) and (16)).

**Step 2 (adjustment)**

The updated particle positions and velocities must be subject to corresponding limits, respectively. Otherwise, they can be adjusted as follows. (1) For *X*_*l*_^1^(*τ*), adjust it to avoid nonrenewable resource infeasibility as shown in Step 2 above. (2) If *x*_*lh*_^2^(*τ*) > 1, then *x*_*lh*_^2^(*τ*) = 1; else if *x*_*lh*_^2^(*τ* + 1) < 0, then *x*_*lh*_^2^(*τ*) = 0. (3) Similarly, if *v*_*lh*_^2^(*τ*) > 1, then *v*_*lh*_^2^(*τ*) = 1; else if *v*_*lh*_^2^(*τ* + 1)<−1, then *v*_*lh*_^2^(*τ*) = −1. After updating and adjustment, the new position *X*_*l*_(*τ*) = [*X*_*l*_^1^(*τ*), *X*_*l*_^2^(*τ*)] is determined.

**Step 3 (particle transformation)**

For each particle *l* (*l* = 1,2,…, *L*), the assignment of modes to activities is determined according to the new position *X*_*l*_^1^(*τ*) in the *τ*th generation. The new position *X*_*l*_^2^(*τ*) is transformed to the scheduling of all activities using the HSGS based on the assignment of modes to activities.

**Step 4 (particle evaluation)**

First calculate the fitness value Fitness (*P*_*l*_(*τ*)) of the particle *l* (*l* = 1,…, *L*) in accordance with the new position *X*_*l*_(*τ*). Then update the personal best of particle *l* using the standard PSO: if Fitness (*X*_*l*_(*τ*)) < Fitness (*P*_*l*_(*τ* − 1)), update *P*_*l*_(*τ*) = *X*_*l*_(*τ*); otherwise, *P*_*l*_(*τ*) = *P*_*l*_(*τ* − 1). Search for the particle with the minimum fitness value and update the global best: update *G*(*τ*) = *P*_*l*_(*τ*), if Fitness(*P*_*l*_(*τ*))<Fitness(*G*(*τ*) − 1). Otherwise, *G*(*τ*) = *G*(*τ* − 1).

**Step 5 (stopping criteria)**

If the stopping criterion is met, that is, *τ* = *T*, go to Step 6. Otherwise, *τ* = *τ* + 1 and return to Step 1.

**Step 6 (decoding)**

Determine the global best position *G* from the particle swarm; then decode the global best position *G* as the solution set.

**Figure 3:**
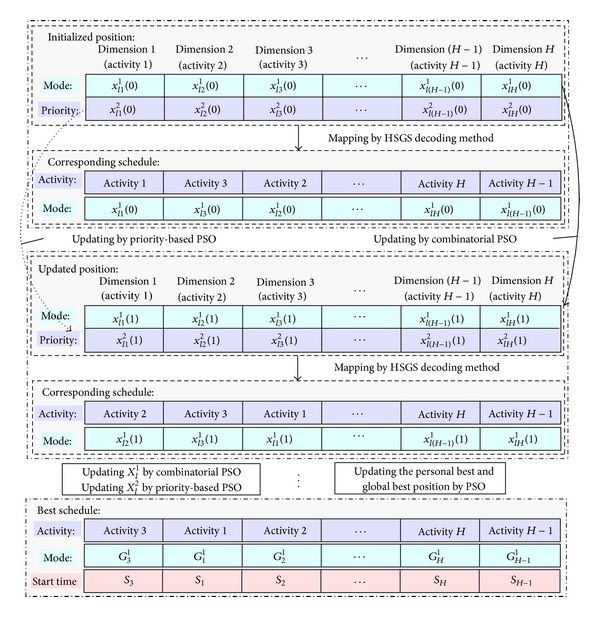
Transformation procedure of the CP-based HPSO.

**Figure 4:**
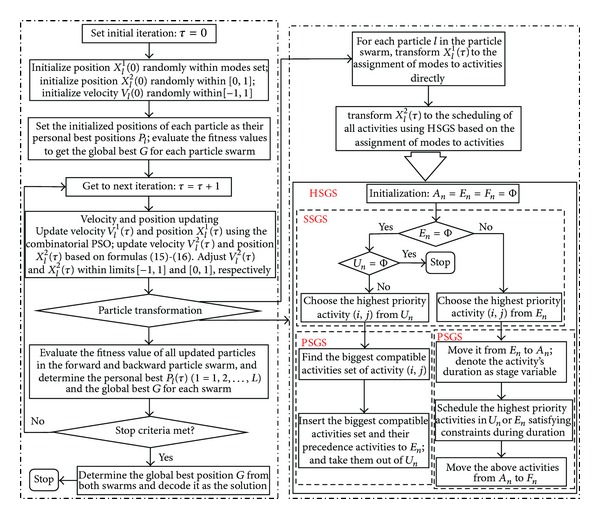
Overall procedure of the CP-based HPSO framework.

Figures 3 and 4 show the transformation and schematic procedure for the CP-based HPSO to generate solutions for MRCMPSP.

## 5. Practical Application to a Construction Project

This section gives a practical application for the proposed MRCMPSP in a large scale water conservancy and hydropower construction project. The case construction procedure contains three projects and two dummy projects (start and end project). Through the illustrative example, the proposed approach is validated and the efficiency of the algorithm is tested.

### 5.1. Project Description

**Figure 5:**
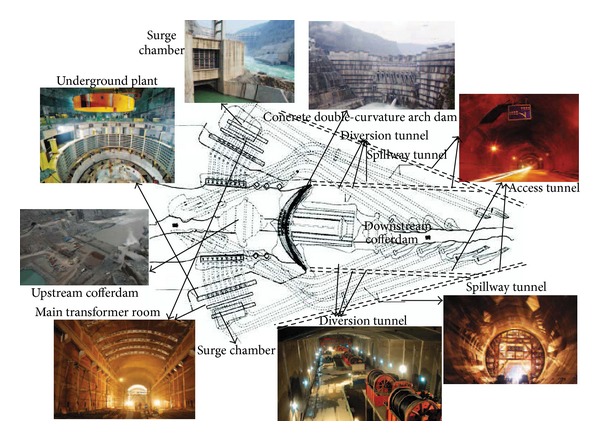
Detailed information of hydropower construction project *X*.

**Figure 6:**
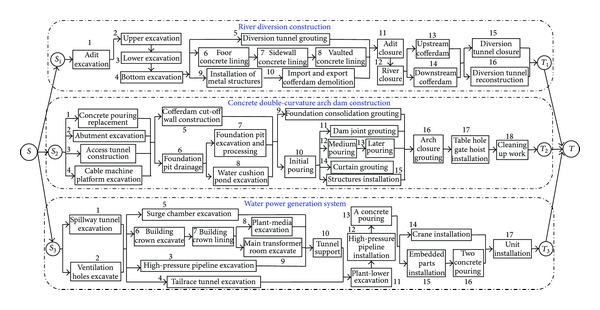
Detailed information of hydropower construction project *X*.

A large scale hydropower construction project (project *X*), located in the southwest region of China, is used as a practical case in this paper and is one of the biggest hydropower projects in China giving rise to many environmental and economic benefits. It has various hydraulic structures including river dam, river diversion, flood discharge structures, and water power generation system. The river dam is a concrete double-curvature arch dam with 278.00 meters high and a dam crest elevation of 610 meters. There are three diversion tunnels on each of the left and right banks. The flood discharge structures consist of four spillway tunnels, seven surface holes, and eight deep holes in the dam, as well as a water cushion pond. The main power house, transformer chamber, and tailrace surge tank in the water power generation system are arranged in parallel. The underground powerhouse has 18 hydroelectric generating sets with a 12,600 MW of installed capacity. This paper focuses on the principal part of hydropower construction project *X*, river diversion construction, concrete double-curvature arch dam construction, and water power generation system construction, the details of which are shown in Figures 5 and 6.

### 5.2. Data Collection and Processing

To collect the related data, site investigations and surveys were conducted to obtain the basic data from both the financial department and the experienced engineers involved with the construction companies, each basic data is with an interval (i.e., [*l*_*q*_, *r*_*q*_]) with the highest possible value (i.e., *m*_*q*_). Then, as shown in Section 2.2, the uncertain parameters are characterized as triangular fuzzy random variables (i.e., ([*ξ*]_*L*_, *φ*(*ω*), [*ξ*]_*R*_), where *φ*(*ω*) ~ *N*(*μ*, *η* ^2^)) based on the collected data and statistical methods, and some relevant data already processed using the above method for the activities are shown in Table 1. Finally, a new method called hybrid crisp approach shown in Section 3.1 is used to convert fuzzy random variables to real numbers. Therefore, all necessary data, including the data converted from the fuzzy random variables based on *σ* = 0.1, *γ* = 0.8, and *λ* = 0.5 and some fixed data, are stated in Tables 2, 3, 4, and 5, among which and for convenience the per-period-availability *R*_*rt*_^*ρ*^ of renewable resource is assumed to be constant *R*_*r*_^*ρ*^.

### 5.3. Parameters Selection for CP-Based HPSO

From the results of the preliminary experiments, which were carried out to observe the behavior of the algorithm at different parameter settings, and through a comparison of several sets of parameters, including population size, iteration number, acceleration constant, initial velocity, and inertia weight, the most suitable parameters were identified.  Table 6 summarizes some of the parameter values selected for the CP-based HPSO in the computational experiments. Note that the population size determines the evaluation runs, which, in turn, impacts the optimization cost, and various learning factors *c*_*p*_ and *c*_*g*_ may lead to small differences in the PSO's performance [53]. The inertia weight *w*(*τ*) is set to be varying with the iterations as shown in (17), and *w*(1) = 0.9 and *w*(*T*) = 0.1 are found to be the most suitable to control the impact of the previous velocities on the current velocity and influence the trade-off between the global and local experiences. The parameter *α* is used to imply intensification and diversification, which induces it to choose the original values or another value.

### 5.4. Computational Results

**Figure 7:**
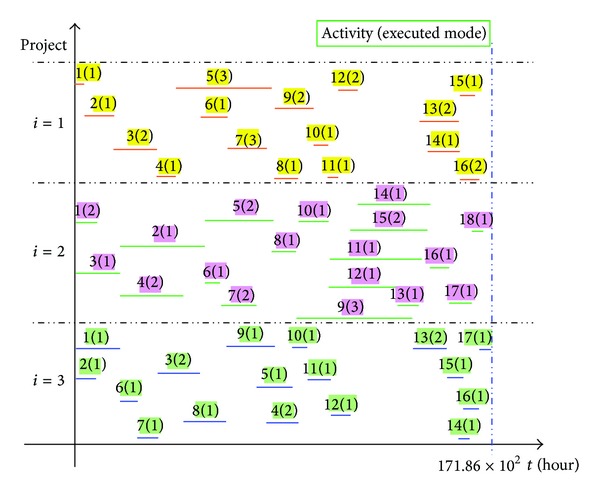
Gantt chart showing the results of the CP-based HPSO algorithm for MRCMPSP in hydropower construction project *X*.

To verify the practicality and efficiency of the optimization method for the MRCMPSP under a fuzzy random environment presented in this paper, the CP-based HPSO is conducted and run on MATLAB 7.0. The computational results, including a satisfactory solution and the multiobjective values, were obtained based on the parameter selection shown in Table 7 (i.e., probability and possibility level, optimistic-pessimistic index, and weights) and the estimated maximal values of *z*_1_, *z*_2_, and *z*_3_ shown in Table 8. The multiobjective values are listed in Table 8, and a satisfactory solution containing mode selection and the start-finish time determination for each activity except the dummy activities is summarized in detail in Table 9.  Figure 7 is a Gantt chart which shows the results of the CP-based HPSO for the MRCMPSP at the hydropower construction project *X*.

### 5.5. Sensitivity Alternative Analysis

In this paper, since there are some undetermined parameters such as the optimistic-pessimistic index *λ*, the probability level *σ*, and possibility level *γ*, the weights between the multiple objectives (i.e., *η*_1_, *η*_2_, and *η*_3_), the weights between the project makespan and project durations (i.e., *λ*_1_, *λ*_2_), and the weights between projects (i.e., *ω*_1_, *ω*_2_, and *ω*_3_), the data for which were provided by project managers and imply the attitude of project managers, further research needs to be done to analyze the sensitivity and advantages compared with other models and algorithms.

#### 5.5.1. Sensitivity Analysis for the Optimistic-Pessimistic Index and Probability-Possibility Levels

The results above were obtained based on the MRCMPSP parameter selection shown in Table 7. As discussed before, there are three uncertain parameters (i.e., the optimistic-pessimistic index *λ*, the probability level *σ*, and the possibility level *γ*) when dealing with fuzzy random variables. It can be seen that, under certain optimistic-pessimistic attitudes and probability-possibility levels, the objective function values are different. To gain further insight into the parameter selection principles, a sensitivity analysis was conducted against these parameters based on the same weights selected above; Table 10 summarizes the different objective function values with respect to the different parameters *λ*, *σ*, and *γ*, where *λ* = 1 and *λ* = 0 are the pessimistic extreme and optimistic extreme, respectively. Based on Section 3.1 and Table 10, the conclusions can be summarized as follows.

1. For the optimistic-pessimistic *λ*, when under the same probability-possibility levels, if *λ* rises, the values stated in Tables 2, 3, and 4 gradually increase, as do the weighted project makespan, project cost, and project quality. This indicates that a more optimistic attitude by the project manager leads to a more optimistic optimization for the weighted project makespan and project cost but with a negative change in the project quality.
2. For the probability level *σ*, under the same optimistic-pessimistic *λ* and possibility level *γ*, when *λ* < 0.5, the bigger *σ*, the bigger the objective function values; when *λ* > 0.5, the bigger *σ*, the smaller the objective function values; when *λ* = 0.5, the change of *σ* has no effect on the objective function values.
3. For the possibility level *γ*, under the same optimistic-pessimistic *λ* and probability level *σ*, when *λ* < 0.5, the bigger *γ*, the smaller the objective function values; when *λ* > 0.5, the bigger *γ*, the bigger the objective function values; when *λ* = 0.5, the change of *γ* depends on the characteristics of the fuzzy random variables themselves.

These results are quite useful and may serve as a reference for decision makers and, in fact, it would be their choice to identify an appropriate set of parameter values to optimize the decision making process. Project managers would be able to fine-tune these parameters to obtain different solutions. These three parameters are provided by the project managers and are interpreted according to the real world problem.

#### 5.5.2. Sensitivity Analysis for the Weights of Objective Functions

From the discussion above, it can be seen that a difference in the weights leads to a difference in the objective function values. The results are shown in Table 11 with respect to the different weights, the optimistic-pessimistic index *λ* = 0.5, the probability level *σ* = 0.1, and the possibility level *γ* = 0.8. These comparative results demonstrate that the difference in the solutions using different weights is not very large, because the weights reflect the importance of each objective from the view of project managers. Therefore, the results become gradually worse with an increase in the importance of the objective function *f*_*w*_. However, in a real situation, project managers would control the weights within a reasonable range and they would be interpreted according to the real world problem.

#### 5.5.3. Model Comparison in Different Environments

To indicate and highlight the superiority of the use of the fuzzy random variables for the mathematical model (14) in this paper, additional computational work was done using the proposed CP-based HPSO to solve the MRCMPSP under another two environments (i.e., a determined environment and a fuzzy environment). In order to guarantee a fair comparison, the related parameters in the MRCMPSP were selected in the following way. Denote the fuzzy random variables as $\tilde{\overset{-}{\xi }}=({\left[\xi \right]}_{L},\varphi (\omega ),{\left[\xi \right]}_{R})$, where *φ*(*ω*) ~ *N*(*μ*, *η* ^2^). Since the variance of *φ*(*ω*) was sufficiently small, and the expectation value *μ* essentially reflected the most possible value over time, it was reasonable to select ([*ξ*]_*L*_, *μ*, [*ξ*]_*R*_) and *μ* as the fuzzy parameter and the certain parameter for a fuzzy environment and a determined environment, respectively. Thus the MRCMPSP models under different environments were formulated and solved using the CP-based HPSO. The computational results obtained based on the MRCMPSP weight selection are shown in Table 12.

By comparing the fitness value of the three objectives and the aggregated objective, the results for the discussed two MRCMPSP types are not better than those for the fuzzy random model, which highlight the superiority of using the fuzzy random variables in the MRCMPSP model in this paper and also indicate that an MRCMPSP model using fuzzy set theory has a much better performance than using certain parameters. The performance also suggests that CP-based HPSO is an effective and relatively efficient approach for solving the MRCMPSP model.

#### 5.5.4. Algorithm Evaluation

To carry out comparisons under similar circumstances, the parameters stated in Table 6 and the initial velocities for the decision variables in the CP-based HPSO were also adopted for the standard PSO.  Table 13 shows the comparison results and the convergence histories of the two types of PSO based on the parameter selection stated in Table 7. From these results, it is obvious that CP-based HPSO has an obvious advantage compared with the standard PSO when solving the MRCMPSP. The first advantage is that the CP-based HPSO is more stable than a standard PSO when searching for the optima. Another advantage is that it is faster when determining the optima and converges a little faster than the standard PSO, that is, the CP-based HPSO needs less iterations to find the optimal solutions. Thus the CP-based HPSO displays an improved search performance compared with the standard PSO under a similar circumstance.

## 6. Conclusions and Future Research

In this paper, a multiobjective optimal control model was established to solve a multimode resource-constrained multiple project scheduling problem (MRCMPSP) in a large scale hydropower construction project under a fuzzy random environment. This is a multiobjective optimization process for minimizing the weighted project makespan and project cost and maximizing the project quality, with decision makers determining suitable project scheduling and mode selection. While using probability theory is cumbersome and costly, and fuzzy theory is incapable of dealing with ambiguous and complex information, triangular fuzzy random variables were used to characterize the multiple parameter uncertainties with combinations of both fuzziness and randomness. A hybrid crisp approach and an expected value operator were introduced to transform these triangular fuzzy random variables to real numbers; thus, the expected value model was derived. Subsequently, to solve the above problem, a multiobjective CP-based HPSO algorithm composed of a priority-based PSO and a combinatorial PSO was developed based on the particular nature of the model, which was able to automatically control the particle-updating in the feasible solution space to find the optimal solution for the expected value model, where the combinatorial PSO was proposed to deal with the selection of modes to activities, and the priority-based PSO was proposed to deal with the scheduling of all activities. Finally, a large scale hydropower construction project composed of a river diversion construction, a concrete double-curvature arch dam construction, and a water power generation system construction was used as a practical application example to verify the maneuverability, scientific nature, advanced nature, and effectiveness of the proposed research. The results and analysis were presented to highlight the performance of our optimization method, which was proven to have the characteristics of generality, reduced calculation time, high velocity, high efficiency, and high precision compared to the standard PSO algorithm.

It should be noted that our MRCMPSP excepted value model was formulated with some assumptions, so it may not represent the precise construction and transportation environment. With this in mind, an important area for future research would be the consideration of more restrictions rather than assumptions. Another area of improvement would be the activity modes being continuous over crashing time rather than being discrete. Therefore, more research needs to be done and evidence gathered in future research to find solutions to the above problems and to develop a more efficient heuristic method to derive modified solutions.

## Figures and Tables

**Table 1:** Detailed information of each activity in the river diversion construction project.

No. of activity	Mode	Nonrenewable resources			Renewable resources		Quality index	Crashed duration
		*k* = 1	*k* = 2	*k* = 3	*r* = 1	*r* = 2		
*S*	1	0	0	0	0	0	0	0
1	1	(10.2, *φ*, 17.6)	(4.4, *φ*, 6.2)	(16.5, *φ*, 19.1)	(4.2, *φ*, 5.6)	(5.2, *φ*, 5.8)	(9.2, *φ*, 9.7)	(2.5, *φ*, 4.9)
		*φ* ~ *N*(13.8,2.3^2^)	*φ* ~ *N*(5.5,0.6^2^)	*φ* ~ *N*(17.7,1.6^2^)	*φ* ~ *N*(4.3,0.7^2^)	*φ* ~ *N*(5.5,0.5^2^)	*φ* ~ *N*(9.4,1.3^2^)	*φ* ~ *N*(3.3,1.0^2^)
	2	(8.5, *φ*, 13.7)	(5.3, *φ*, 7.6)	(14.1, *φ*, 17.4)	(3.6, *φ*, 4.7)	(3.6, *φ*, 5.7)	(8.8, *φ*, 9.3)	(2.8, *φ*, 5.7)
		*φ* ~ *N*(10.9,3.6^2^)	*φ* ~ *N*(6.2,0.8^2^)	*φ* ~ *N*(15.8,2.0^2^)	*φ* ~ *N*(4.1,0.8^2^)	*φ* ~ *N*(4.3,0.8^2^)	*φ* ~ *N*(9.0,1.7^2^)	*φ* ~ *N*(3.9,1.1^2^)
2	1	(35.5, *φ*, 38.7)	(12.8, *φ*, 17.3)	(23.3, *φ*, 26.5)	(8.3, *φ*, 11.5)	(6.2, *φ*, 8.4)	(9.4, *φ*, 9.7)	(9.8, *φ*, 14.7)
		*φ* ~ *N*(37.1,3.0^2^)	*φ* ~ *N*(15.6,2.0^2^)	*φ* ~ *N*(25.1,2.1^2^)	*φ* ~ *N*(9.6,1.2^2^)	*φ* ~ *N*(7.3,1.0^2^)	*φ* ~ *N*(9.5,1.8^2^)	*φ* ~ *N*(12.6,2.0^2^)
	2	(31.3, *φ*, 36.5)	(10.7, *φ*, 13.5)	(25.2, *φ*, 29.1)	(6.3, *φ*, 8.0)	(6.5, *φ*, 7.2)	(8.9, *φ*, 9.5)	(11.0, *φ*, 14.3)
		*φ* ~ *N*(33.2,2.5^2^)	*φ* ~ *N*(12.6,2.3^2^)	*φ* ~ *N*(27.4,2.1^2^)	*φ* ~ *N*(7.5,1.1^2^)	*φ* ~ *N*(6.8,1.0^2^)	*φ* ~ *N*(9.1,1.6^2^)	*φ* ~ *N*(12.0,2.2^2^)
⋮								
16	1	(32.3, *φ*, 35.5)	(22.6, *φ*, 27.3)	(26.2, *φ*, 32.5)	(7.2, *φ*, 9.3)	(5.2, *φ*, 7.6)	(9.5, *φ*, 9.8)	(5.5, *φ*, 8.3)
		*φ* ~ *N*(33.6,2.0^2^)	*φ* ~ *N*(24.7,2.1^2^)	*φ* ~ *N*(28.8,1.7^2^)	*φ* ~ *N*(8.1,1.0^2^)	*φ* ~ *N*(6.3,1.1^2^)	*φ* ~ *N*(9.6,1.3^2^)	*φ* ~ *N*(6.9,1.6^2^)
	2	(28.4, *φ*, 31.5)	(23.3, *φ*, 27.6)	(24.2, *φ*, 27.4)	(6.5, *φ*, 8.7)	(4.1, *φ*, 6.5)	(8.9, *φ*, 9.3)	(7.2, *φ*, 9.0)
		*φ* ~ *N*(29.9,2.1^2^)	*φ* ~ *N*(25.4,1.8^2^)	*φ* ~ *N*(25.6,1.8^2^)	*φ* ~ *N*(7.3,1.2^2^)	*φ* ~ *N*(5.4,0.7^2^)	*φ* ~ *N*(9.1,1.2^2^)	*φ* ~ *N*(8.2,1.3^2^)
*T*	1	0	0	0	0	0	0	0

No. of activity		Normal duration	Unit indirect cost	Unit crashing cost	Fixed direct cost	Earned value	Predecessors	

*S*		0	0	0	0	0		
1		(5.2, *φ*, 6.6)	(20.7, *φ*, 23.0)	(15.2, *φ*, 17.4)	39.5	87.6		
		*φ* ~ *N*(5.9,1.2^2^)	*φ* ~ *N*(21.3,2.1^2^)	*φ* ~ *N*(16.1,3.6^2^)				
2		(13.0, *φ*, 16.5)	(22.6, *φ*, 25.5)	(26.8, *φ*, 30.8)	85.6	123.7	1	
		*φ* ~ *N*(14.9,2.3^2^)	*φ* ~ *N*(24.3,2.0^2^)	*φ* ~ *N*(28.3,3.2^2^)				
⋮								
16		(8.2, *φ*, 10.6)	(16.3, *φ*, 19.7)	(36.8, *φ*, 39.2)	46.2	123.1	13,14	
		*φ* ~ *N*(9.5,1.5^2^)	*φ* ~ *N*(18.2,2.1^2^)	*φ* ~ *N*(37.9,2.9^2^)				
*T*		0	0	0	0	0		

**Table 2:** Detailed information of each activity in the river diversion construction project.

(1, *j*)	*M*_1*j*_	*r*_1*jk*_^*m*^			*r*_1*jr*_^*m*^		*q*_1*j*_^*m*^	*d*_1*j*_^*m*^	*d*_1*j*_	*c*_1*j*_	*k*_1*j*_	*C*_1*j*_	*EV*_1*j*_	*P*_1*j*_
		*k* = 1	*k* = 2	*k* = 3	*r* = 1	*r* = 2								
		(10^4^)	(10^4^)	(10^4^)	(10^3^)	(10^2^)		(10^2^)	(10^2^)	(10^6^)	(10^6^)	(10^6^)		
*S*	1	0	0	0	0	0	0	0	0	0	0	0	0	
1	1	13.86	5.38	17.76	4.66	5.50	9.43	3.54	5.90	21.63	16.22	39.5	87.6	S
	2	11.02	6.35	15.77	4.13	4.51	9.03	4.11						
2	1	37.10	15.27	24.98	9.78	7.30	9.53	12.39	14.81	24.15	28.60	85.6	123.7	1
	2	33.62	12.30	27.25	7.29	6.83	9.16	13.07						
3	1	40.20	21.56	30.00	10.15	7.86	9.81	16.46	19.33	27.82	36.53	98.2	165.8	2
	2	43.52	18.90	28.13	9.30	8.61	9.42	18.05						
	3	46.69	17.71	27.52	8.16	8.13	9.05	18.52						
4	1	27.78	10.65	20.14	7.12	6.73	9.65	7.96	9.15	18.84	39.77	65.3	116.5	3
5	1	16.02	35.68	82.36	7.81	8.20	9.60	37.11	41.86	10.30	35.64	132.6	213.4	4
	2	13.15	32.35	75.73	7.26	6.79	8.95	39.14						
	3	12.84	32.35	79.10	6.79	7.68	9.26	39.65						
6	1	24.25	87.36	12.14	7.71	8.72	9.84	10.80	14.85	18.30	20.18	121.7	176.4	4
	2	27.15	83.90	11.12	6.35	7.50	9.06	13.63						
7	1	34.38	97.42	17.83	8.65	9.00	9.35	15.10	19.38	23.25	31.83	148.2	234.5	6
	2	28.16	93.93	15.25	7.01	7.28	8.92	17.61						
	3	30.92	91.84	14.10	7.70	7.80	9.13	16.28						
8	1	20.15	60.20	10.62	7.65	7.34	9.40	9.91	12.74	17.20	23.62	105.3	167.8	7
	2	22.86	63.25	8.79	6.57	6.83	9.63	10.86						
9	1	0	0	0	12.85	7.15	9.52	14.76	18.16	18.25	31.85	135.6	191.2	4
	2	0	0	0	10.45	5.75	9.01	16.11						
10	1	0	0	0	11.42	7.78	9.52	5.84	8.68	18.14	20.18	48.7	105.7	9
	2	0	0	0	9.57	7.23	8.79	7.15						
11	1	17.28	5.86	8.30	4.17	3.92	9.37	3.48	4.61	10.24	28.25	26.4	94.3	5, 8, 10
12	1	79.45	9.05	25.31	7.37	6.28	9.56	7.05	10.46	23.20	31.66	86.3	155.2	11
	2	75.93	8.25	27.19	6.52	5.12	9.05	8.14						
	3	82.78	7.84	30.58	6.16	5.40	9.73	9.20						
13	1	179.36	22.21	65.85	11.83	9.15	9.87	14.25	18.75	26.70	39.15	83.7	215.1	12
	2	172.72	20.06	69.63	9.45	8.81	9.32	16.11						
14	1	171.74	20.15	52.16	11.46	9.85	9.64	13.28	17.12	25.74	36.57	80.5	198.3	12
	2	163.27	17.61	55.81	10.03	8.13	9.43	15.50						
15	1	39.75	8.08	18.62	6.50	5.73	9.70	6.02	7.86	12.54	25.62	52.5	118.3	13, 14
16	1	33.78	24.85	29.13	8.19	6.36	9.63	6.90	9.44	18.08	37.96	46.2	123.1	13, 14
	2	29.93	25.43	25.72	7.48	5.34	9.10	8.14						
*T*	1	0	0	0	0	0	0	0	0	0	0	0	0	15, 16

**Table 3:** Detailed information of each activity in the concrete double-curvature arch dam construction project.

(2, *j*)	*M*_2*j*_	*r*_2*jk*_^*m*^			*r*_2*jr*_^*m*^		*q*_2*j*_^*m*^	*d*_2*j*_^*m*^	*d*_2*j*_	*c*_2*j*_	*k*_2*j*_	*C*_2*j*_	*EV*_2*j*_	*P*_2*j*_
		*k* = 1	*k* = 2	*k* = 3	*r* = 1	*r* = 2								
		(10^4^)	(10^4^)	(10^4^)	(10^3^)	(10^2^)		(10^2^)	(10^2^)	(10^6^)	(10^6^)	(10^6^)		
*S*	1	0	0	0	0	0	0	0	0	0	0	0	0	
1	1	18.03	35.74	26.15	5.24	5.02	9.61	8.03	11.05	16.33	28.35	43.5	118.5	*S*
	2	16.47	38.38	23.43	4.50	4.15	9.21	8.85						
2	1	62.78	69.84	38.06	13.65	10.50	9.50	35.40	41.15	28.20	30.12	164.2	216.7	*S*
	2	62.25	66.41	34.18	11.31	9.02	8.86	37.15						
	3	60.83	68.15	35.46	10.73	10.14	9.05	38.32						
3	1	27.16	42.52	37.60	8.26	9.84	9.38	18.41	22.17	26.35	27.21	76.4	172.5	*S*
	2	25.37	46.15	34.28	7.52	8.05	8.79	20.56						
4	1	44.13	51.10	29.85	12.46	10.53	9.22	24.15	28.70	22.76	36.81	65.3	189.8	*S*
	2	41.35	52.56	27.18	10.62	9.06	9.73	26.37						
5	1	46.45	28.60	26.73	9.16	8.75	9.74	26.15	31.04	16.72	28.53	84.5	208.6	1, 2, 3, 4
	2	49.74	25.67	20.16	8.02	7.31	9.28	28.67						
6	1	0	0	0	5.62	3.28	9.75	6.37	7.82	10.51	23.45	31.4	105.7	1, 2, 3, 4
7	1	23.26	38.36	14.32	8.03	4.79	9.50	13.45	18.02	18.16	35.61	78.7	165.8	6
	2	28.14	35.52	10.56	7.00	3.56	9.07	14.69						
8	1	20.50	17.82	27.45	6.80	8.27	9.60	10.27	12.84	15.14	21.74	65.7	108.3	6
	2	23.51	20.73	21.38	6.02	8.16	9.15	11.04						
9	1	18.15	37.25	97.22	8.36	6.46	9.61	46.38	52.26	16.90	27.14	176.5	216.2	5, 7, 8
	2	16.57	38.31	93.28	6.25	5.58	8.90	49.36						
	3	15.04	35.68	94.13	7.10	7.49	9.29	48.50						
10	1	12.62	49.55	15.74	10.74	12.65	9.71	12.39	17.55	25.00	28.60	123.5	189.7	5, 7, 8
	2	10.41	46.92	13.58	9.66	10.83	9.27	15.58						
11	1	13.65	30.52	65.47	7.72	5.35	9.58	38.41	43.18	12.72	15.30	115.2	178.5	10
	2	12.08	27.15	62.75	5.39	4.80	9.23	41.24						
12	1	34.52	123.66	35.55	10.84	11.20	9.85	28.33	33.76	29.53	28.02	256.4	315.6	10
	2	36.18	120.17	33.27	9.74	10.58	9.20	30.46						
	3	32.45	115.36	30.59	10.52	9.16	8.89	32.30						
13	1	15.48	51.35	18.66	9.92	11.06	9.55	8.48	11.38	33.09	38.65	90.3	182.7	12
	2	16.73	48.08	16.25	8.14	9.60	9.13	10.05						
14	1	16.35	33.74	70.50	6.31	5.08	9.67	30.27	33.13	13.26	11.12	121.3	223.4	10
	2	16.35	31.06	66.75	5.42	4.74	9.11	31.86						
15	1	0	0	0	9.85	6.30	9.81	31.26	34.59	17.56	27.84	113.6	206.5	10
	2	0	0	0	8.72	6.86	9.43	32.39						
16	1	8.25	13.58	39.16	8.68	7.65	9.74	8.14	8.90	14.25	27.66	86.2	137.5	9, 11, 13, 14, 15
17	1	0	0	0	8.53	8.06	9.78	9.20	10.06	20.17	52.25	76.1	115.7	16
18	1	0	0	0	10.86	4.15	9.54	4.60	5.18	12.10	27.64	35.3	93.0	17
*T*	1	0	0	0	0	0	0	0	0	0	0	0	0	18

**Table 4:** Detailed information of each activity in the water power generation system project.

(3, *j*)	*M*_3*j*_	*r*_3*jk*_^*m*^			*r*_3*jr*_^*m*^		*q*_3*j*_^*m*^	*d*_3*j*_^*m*^	*d*_3*j*_	*c*_3*j*_	*k*_3*j*_	*C*_3*j*_	*EV*_3*j*_	*P*_3*j*_
		*k* = 1	*k* = 2	*k* = 3	*r* = 1	*r* = 2								
		(10^4^)	(10^4^)	(10^4^)	(10^3^)	(10^2^)		(10^2^)	(10^2^)	(10^6^)	(10^6^)	(10^6^)		
*S*	1	0	0	0	0	0	0	0	0	0	0	0	0	
1	1	25.22	40.20	32.72	9.75	8.88	9.46	18.41	21.57	24.16	19.50	78.6	162.3	*S*
	2	22.19	42.85	30.16	8.66	7.04	9.11	19.84						
2	1	16.30	28.45	21.36	8.63	5.82	9.59	8.32	10.67	20.58	42.14	62.5	145.7	*S*
3	1	32.53	26.55	38.16	9.73	9.65	9.60	16.14	18.70	18.91	35.34	93.7	194.8	1, 2
	2	35.74	24.03	35.28	8.65	8.72	9.18	17.70						
4	1	28.65	35.62	25.82	8.36	8.37	9.15	11.37	14.72	22.45	30.61	66.8	156.5	1, 2
	2	26.49	39.25	23.51	7.48	7.16	8.73	13.45						
5	1	39.70	38.32	27.85	7.37	4.55	9.72	15.04	18.57	18.65	23.50	102.3	215.2	1, 2
	2	33.17	35.27	30.15	5.73	4.14	9.38	16.78						
6	1	17.52	14.75	15.94	6.50	5.78	9.82	7.26	8.51	20.76	32.00	89.6	138.6	1, 2
7	1	14.39	47.52	10.25	7.13	6.57	9.64	8.67	10.02	28.10	37.43	95.7	150.8	6
8	1	46.48	36.83	33.13	10.63	8.73	9.60	17.70	20.92	27.64	21.75	118.6	209.5	7
	2	41.35	40.00	30.56	9.81	8.27	9.00	19.14						
9	1	58.95	40.72	36.35	11.07	8.57	9.14	20.18	24.70	27.69	20.15	124.7	217.0	7
	2	53.21	38.46	37.62	9.72	8.25	9.53	21.71						
	3	56.10	34.97	32.54	8.56	8.25	8.74	23.16						
10	1	4.13	8.05	7.22	8.30	5.16	9.75	6.37	8.34	27.48	56.53	92.4	110.5	3, 5, 8, 9
11	1	24.72	18.43	18.15	10.46	8.02	9.55	9.38	13.52	18.29	21.75	96.3	149.4	4, 10
	2	27.11	16.85	15.66	8.35	7.16	9.21	12.84						
12	1	12.26	45.63	3.28	8.80	6.84	9.35	8.14	11.13	27.55	20.18	77.5	176.3	11
	2	15.65	40.87	2.66	7.47	6.07	9.58	9.72						
13	1	20.37	56.50	8.75	9.83	8.41	9.28	15.23	19.12	24.68	30.35	131.1	245.9	12
	2	18.15	59.12	7.60	8.77	7.54	9.50	13.98						
	3	22.09	52.74	7.06	8.25	6.88	8.83	16.75						
14	1	0	0	0	8.83	7.62	9.75	4.24	5.73	18.36	38.22	69.2	106.5	13
15	1	5.27	4.30	7.32	9.57	7.25	9.64	6.36	8.72	24.65	16.73	83.7	124.6	13
	2	4.15	3.71	6.25	8.34	6.38	9.12	7.28						
16	1	13.15	26.52	4.36	9.66	7.16	9.18	6.20	8.39	24.52	37.37	78.8	136.7	15
	2	10.64	28.11	3.56	9.07	6.52	9.43	7.41						
17	1	16.58	10.29	21.58	16.62	6.48	9.73	4.96	7.83	43.18	53.26	137.4	231.5	14, 16
	2	15.14	8.65	22.71	15.18	5.30	9.16	6.29						
*T *	1	0	0	0	0	0	0	0	0	0	0	0	0	17

**Table 5:** Some fixed data in hydropower construction project *X*.

*T*	*B*	*R*_1_^*υ*^	*R*_2_^*υ*^	*R*_3_^*υ*^	*R*_1_^*ρ*^	*R*_2_^*ρ*^
(10^2^ hour)	(10^8^ RMB)	(10^4^ m^3^)	(10^4^ m^3^)	(10^4^ t)	(10^3^)	(10^2^)
200.64	248.98	1476.83	1516.46	1254.71	41.00	36.00

**Table 6:** Parameters selection for the proposed CP-based HPSO.

Population size	Iteration number	Acceleration constant		Inertia weight		Parameter
*L*	*T*	*c*_*p*_	*c*_*g*_	*w*(1)	*w*(*T*)	*α*
100	150	2	2	0.9	0.1	0.3

**Table 7:** Parameters selection for MRCMPSP.

*σ*	*γ*	*λ*	*η*_1_	*η*_2_	*η*_3_	*λ*_1_	*λ*_2_	*ω*_1_	*ω*_2_	*ω*_3_
0.1	0.8	0.5	035	0.35	0.30	0.60	0.40	0.25	0.40	0.35

**Table 8:** Results of multiple objectives values using the CP-based HPSO algorithm for MRCMPSP.

*z*	*z*_1_	*z*_2_	*z*_3_	*z*_1_^max^	*z*_2_^max^	*z*_3_^max^
	(10^2^ hour)	(10^6^ RMB)		(10^2^ hour)	(10^6^ RMB)	
0.3471	170.8878	24180.1635	9.4789	200.6400	24569.6824	9.6241

**Table 9:** Results of the CP-based HPSO algorithm for MRCMPSP.

*i* = 1					*i* = 2					*i* = 3				
*j*	*m*	*S*_*ij*_	*d*_*ij*_^*m*^	*F*_*ij*_	*j*	*m*	*S*_*ij*_	*d*_*ij*_^*m*^	*F*_*ij*_	*j*	*m*	*S*_*ij*_	*d*_*ij*_^*m*^	*F*_*ij*_
		(10^2^)	(10^2^)	(10^2^)			(10^2^)	(10^2^)	(10^2^)			(10^2^)	(10^2^)	(10^2^)
1	1	0	3.54	3.54	1	2	0	8.85	8.85	1	1	0	18.41	18.41
2	1	3.54	12.39	15.93	2	1	18.41	35.40	53.81	2	1	0	8.32	8.32
3	2	15.93	18.05	33.98	3	1	0	18.41	18.41	3	2	34.34	17.70	52.04
4	1	33.98	7.96	41.94	4	2	18.41	26.37	44.78	4	2	79.12	13.45	92.57
5	3	41.94	39.65	81.59	5	2	53.81	28.67	82.48	5	1	74.87	15.04	89.91
6	1	52.04	10.80	62.84	6	1	53.81	6.37	60.18	6	1	18.41	7.26	25.67
7	3	62.84	16.28	79.12	7	2	60.18	14.69	74.87	7	1	25.67	8.67	34.34
8	1	82.48	9.91	92.39	8	1	81.59	10.27	91.86	8	1	44.78	17.70	62.48
9	2	82.66	16.11	98.77	9	3	91.86	48.5	140.36	9	1	62.48	20.18	82.66
10	1	98.77	5.84	104.61	10	1	92.57	12.39	104.96	10	1	89.91	6.37	96.28
11	1	104.61	3.48	108.09	11	1	104.96	38.41	143.37	11	1	96.28	9.38	105.66
12	2	108.09	8.14	116.23	12	1	104.96	28.33	133.29	12	1	105.66	8.14	113.80
13	2	141.77	16.11	157.88	13	1	133.29	8.48	141.77	13	2	140.36	13.98	154.34
14	1	146.19	13.28	159.47	14	1	116.23	30.27	146.50	14	1	157.88	4.24	162.12
15	1	159.47	6.02	165.49	15	2	113.80	32.39	146.19	15	1	154.34	6.36	160.70
16	2	159.47	8.14	167.61	16	1	146.50	8.14	154.64	16	1	160.70	6.20	166.90
					17	1	154.64	9.20	163.84	17	1	166.90	4.96	171.86
					18	1	163.84	4.60	168.44					

**Table 10:** Sensitivity analysis on optimistic-pessimistic index and probability-possibility levels.

*λ*	Objective	*σ* = 0.025			*σ* = 0.05			*σ* = 0.075			*σ* = 0.1		
		*γ* = 0.7	*γ* = 0.8	*γ* = 0.9	*γ* = 0.7	*γ* = 0.8	*γ* = 0.9	*γ* = 0.7	*γ* = 0.8	*γ* = 0.9	*γ* = 0.7	*γ* = 0.8	*γ* = 0.9
0.00	*z*	0.3269	0.3230	0.3180	0.3321	0.3277	0.3225	0.3373	0.3302	0.3271	0.3415	0.3364	0.3315
0.25	*z*	0.3408	0.3364	0.3327	0.3437	0.3393	0.3358	0.3462	0.3427	0.3392	0.3485	0.3455	0.3418
0.50	*z*	0.3502	0.3471	0.3508	0.3502	0.3471	0.3508	0.3502	0.3471	0.3508	0.3502	0.3471	0.3508
0.75	*z*	0.3603	0.3645	0.3684	0.3572	0.3613	0.3648	0.3550	0.3587	0.3620	0.3531	0.3562	0.3597
1.00	*z*	0.3710	0.3763	0.3816	0.3685	0.3735	0.3771	0.3663	0.3700	0.3742	0.3637	0.3675	0.3708

**Table 11:** Sensitivity analysis on the weights selection by project managers.

(*η*_1_, *η*_2_, *η*_3_)	Fitness	*ω*_1_ = 0.2, *ω*_2_ = 0.4, *ω*_3_ = 0.4			*ω*_1_ = 0.25, *ω*_2_ = 0.4, *ω*_3_ = 0.35			*ω*_1_ = 0.3, *ω*_2_ = 0.35, *ω*_3_ = 0.35		
	Value	*λ*_1_ = 0.4	*λ*_1_ = 0.5	*λ*_1_ = 0.6	*λ*_1_ = 0.4	*λ*_1_ = 0.5	*λ*_1_ = 0.6	*λ*_1_ = 0.4	*λ*_1_ = 0.5	*λ*_1_ = 0.6
	Function	*λ*_2_ = 0.6	*λ*_2_ = 0.5	*λ*_2_ = 0.4	*λ*_2_ = 0.6	*λ*_2_ = 0.5	*λ*_2_ = 0.4	*λ*_2_ = 0.6	*λ*_2_ = 0.5	*λ*_2_ = 0.4
(0.40, 0.35, 0.25)	*z*	0.4550	0.4458	0.4378	0.4566	0.4473	0.4389	0.4577	0.4481	0.4393
(0.40, 0.30, 0.30)	*z*	0.3542	0.3457	0.3393	0.3556	0.3473	0.3405	0.3568	0.3482	0.3409
(0.35, 0.40, 0.25)	*z*	0.4673	0.4549	0.4445	0.4689	0.4563	0.4456	0.4701	0.4571	0.4460
(0.35, 0.35, 0.30)	*z*	0.3612	0.3540	0.3460	0.3628	0.3556	0.3471	0.3640	0.3565	0.3475
(0.35, 0.30, 0.35)	*z*	0.2706	0.2600	0.2476	0.2721	0.2613	0.2487	0.2734	0.2621	0.2491
(0.30, 0.40, 0.30)	*z*	0.3750	0.3643	0.3528	0.3765	0.3658	0.3537	0.3775	0.3666	0.3542
(0.30, 0.35, 0.35)	*z*	0.2781	0.2655	0.2543	0.2795	0.2671	0.2553	0.2806	0.2680	0.2557

**Table 12:** Model comparison in different environments.

Environments	Objective function values			
	*z*	*z*_1_ (10^2^)	*z*_2_ (10^6^)	*z*_3_
Determined environment	0.3727	192.6145	24316.8100	9.6315
Fuzzy environment	0.3578	176.2357	24205.6548	9.5126
Fuzzy random environment	0.3471	170.8878	24180.1635	9.4789

**Table 13:** Comparison results between CP-based HPSO and standard PSO.

Algorithm	Objective function values				Convergence iteration number	Computation time (s)
	*z*	*z*_1_ (10^2^)	*z*_2_ (10^6^)	*z*_3_		
CP-based HPSO	0.3471	170.8878	24180.1635	9.4789	58	26.3264
Standard PSO	0.3471	170.8878	24180.1635	9.4789	124	57.1683
